# Prevalence and Factors Associated with the Desire to Avoid Pregnancy in Never-Pregnant Patients with Systemic Lupus Erythematosus

**DOI:** 10.3390/jcm14186394

**Published:** 2025-09-10

**Authors:** Worawit Louthrenoo, Wanitcha Gumtorntip, Nuntana Kasitanon, Kajohnsak Noppakun, Napatra Tovanabutra, Antika Wongthanee

**Affiliations:** 1 Division of Rheumatology, Department of Internal Medicine, Faculty of Medicine, Chiang Mai University, Chiang Mai 50200, Thailand; wanitcha.ch@cmu.ac.th (W.G.); nkasitan@gmail.com (N.K.); 2 Division of Nephrology, Department of Internal Medicine, Faculty of Medicine, Chiang Mai University, Chiang Mai 50200, Thailand; kajohnsak.noppakun@cmu.ac.th; 3 Pharmacoepidemiology and Statistics Research Center, Faculty of Pharmacy, Chiang Mai University, Chiang Mai 50200, Thailand; 4 Division of Dermatology, Department of Internal Medicine, Faculty of Medicine, Chiang Mai University, Chiang Mai 50200, Thailand; napatra.to@cmu.ac.th; 5 Research Unit, Department of Internal Medicine, Faculty of Medicine, Chiang Mai University, Chiang Mai 50200, Thailand; antika.wong@cmu.ac.th

**Keywords:** systemic lupus erythematosus, pregnancy, conception

## Abstract

**Objective:** To determine the prevalence and factors associated with the desire to avoid pregnancy in Thai patients with systemic lupus erythematosus (SLE). **Methods:** All female SLE patients (aged 18–50 years, menstruating, and never-pregnant) seen between November 2021 and July 2023 at Chiang Mai University Hospital were invited to participate in this cross-sectional questionnaire survey. The exclusion criteria were female patients with female partners and patients with overlapping syndromes. The participants were asked to rate their perception of SLE severity based on organ involvement and to indicate whether they wished to be pregnant. **Results:** Among the 201 participants (mean age 30.75 ± 7.77 years, median disease duration 9.31 years, single 73.37%, and tertiary level of education 85.50%), 127 (63.18%) desired to avoid pregnancy, with the reasons being personal decision in 72 (56.69%), fear of disease worsening in 60 (47.24%), past negative experience in 60 (47.24%), concern about fetal effects of disease or medications in 31 (24.41%), and physicians’ recommendation in 10 (7.87%). Age, marriage or living with a partner, and having a tertiary level of education were independent factors associated with avoiding pregnancy, with adjusted odds ratios and 95% confidence intervals (AOR [95% CI]) of 1.07 (1.02–1.11), *p* = 0.005; 0.387 (0.18–0.77), *p* = 0.008 and 0.29 (0.10–0.81), *p* = 0.018, respectively. **Conclusions:** Over half of the Thai never-pregnant patients with SLE did not wish to become pregnant. Increasing age was an independent factor associated with this decision, while being married or living with a partner, as well as having a high level of education (tertiary education), were independent factors associated with less likelihood of avoiding pregnancy. Understanding why SLE patients of childbearing age do not wish to become pregnant could lead to greater flexibility in managing severe SLE disease.

## 1. Introduction

Systemic lupus erythematosus (SLE) is a chronic autoimmune condition characterized by episodes of remission and relapse that affect multiple organs in the body. The disease most commonly occurs in females of reproductive age. Given this, female SLE patients with male partners are more likely to want their own children. However, pregnancy in SLE poses considerable clinical challenges, due to the complex interplay between disease activity, therapeutic interventions, and pregnancy outcomes. Several studies and reviews have shown that active SLE during pregnancy is associated with elevated risks of maternal and fetal complications, including disease flares, preeclampsia, prematurity, and adverse perinatal outcome [1,2,3,4,5,6].

Management of SLE often necessitates the use of glucocorticosteroids and immunosuppressive agents [7,8] to control the disease. Several immunosuppressive agents, including methotrexate, mycophenolate mofetil, cyclophosphamide, azathioprine, cyclosporine, tacrolimus, belimumab, and anifrolumab are available for treating SLE [7,8,9,10]. However, some of these medications, notably methotrexate, mycophenolate mofetil, and cyclophosphamide are potentially teratogenic and thus contraindicated during pregnancy [10,11,12]. In particular, cyclophosphamide is often reserved for severe or life-threatening SLE manifestations, including renal, neuropsychiatric, cardiopulmonary, or systemic vasculitis involvement [7,8,13,14], but its use can result in premature ovarian failure, resulting in the inability to conceive [15,16].

In addition, an SLE patient who plans to become pregnant should be in a period of sustained remission or low disease activity for at least 6 months prior to conception [6,10,11,17]. The decision to conceive or avoid pregnancy thus requires careful consideration of both disease status and treatment regimen. Furthermore, patients with SLE are occasionally hospitalized due to the severity of the disease, as well as complications arising from its treatment [18]. These factors may discourage women with SLE from wanting to become pregnant. Therefore, understanding whether a woman with SLE wishes to avoid pregnancy is crucial in managing patients with highly active disease, as this could lead to greater flexibility in treating the patients with immunosuppressive drugs that are potentially teratogenic.

This study was carried out to determine the prevalence and factors associated with the desire to avoid pregnancy in female Thai patients with SLE. While those who are pregnant might desire to have children, and those who have had them might not want more, a potential bias could arise when assessing the wish to avoid pregnancy. Therefore, this study only included SLE patients who had never been pregnant.

## 2. Materials and Methods

### 2.1. Patients

All female Thai SLE out-patients attending the Rheumatology, Nephrology, and Dermatology clinics of Chiang Mai University Hospital between November 2021 and July 2023 were invited to join this cross-sectional questionnaire survey. The inclusion criteria comprised age 18–50 years, current menstruating, and never been pregnant. The exclusion criteria were female patients with female partners, and patients having overlapping syndrome with other rheumatic diseases (such as systemic sclerosis, vasculitis syndrome, polymyositis, rheumatoid arthritis, etc.). Past medical history was gathered from data available in medical records. SLE was diagnosed according to the 1997 American College of Rheumatology (ACR) Updating Criteria for Classification of Systemic Lupus Erythematosus [19], or the 2012 Systemic Lupus Erythematosus International Collaboration Clinics (SLICC) classification criteria for systemic lupus erythematosus [20]. SLE disease activity was determined by Systemic Lupus Erythematosus Disease Activity Index (SLEDAI) score, but only the clinical part (cSLEDAI) was used for analysis [21]. Remission was defined according to the 2021 International Task Force (Definition Of Remission In SLE: DORIS), but physician global assessment (PGA) score was not included, due to it not being routinely recorded [22]. Organ damage was determined by the SLICC/ACR Damage Index (SDI) [23].

### 2.2. The Questionnaire

The questionnaire was developed by 3 authors (WL, WG, and NK) and a nurse (WS). It consisted of two parts. Part I (12 questions) related to the participants’ demographic characteristics, marital status, educational level, and their perception of SLE severity based on their personal experience (e.g., organ involvement, medications received, or hospitalization). Part II (4 questions) focused on the desire to become pregnant or avoid pregnancy, as well as the reasons for wanting to avoid pregnancy (e.g., fear of potential complications to the fetus, perception of having severe diseases, or prior experiences such as previous illness or hospitalization). Pilot testing and cognitive debriefing were performed with 5 SLE patients and one schoolteacher. After correcting the wording, grammar, and typographical errors, the final version of the questionnaire was developed for use. However, the patients were allowed not to answer questions that they considered too sensitive or personal.

### 2.3. Sample Size Calculation

A study from the Netherlands reported a 41% prevalence of childlessness in SLE patients [24]. Assuming that the avoidance of pregnancy among Thai SLE patients varied by 10% from that of the Netherlands, this study required a sample size of at least 199 patients. This calculation was made with a test power of 80% and a 95% confidence interval (95% CI).

### 2.4. Ethical Approval

This study was approved by the Ethics Committee of the Faculty of Medicine, Chiang Mai University (No. 437/2021), and conducted according to the guidelines of the Declaration of Helsinki. All of the participants provided their written informed consent prior to entering the study.

### 2.5. Statistical Analysis

STATA 16.0 for Windows (StataCorp LLC, College Station, TX, USA) was used to perform statistical analysis. Continuous variables were presented as mean ± standard deviation (SD) or median (interquartile range, Q1–Q3) where appropriate. Categorical variables were described as numbers or percentages. Student’s *t*-test or Mann–Whitney U test was used for comparison of continuous variables depending on distribution of the data, whereas Chi^2^ or Fisher’s Exact test was used to determine the difference among the categorical variables. Variables with a *p*-value of <0.15 in the univariable analysis, as well as any variables deemed clinically significant, were included in the multivariable logistic regression analysis. Backward selection was used to remove variables with a significance level >0.15 from the model, in order to identify independent factors associated with the desire to avoid pregnancy. A *p*-value of <0.05 was considered statistically significant.

## 3. Results

### 3.1. Demographic Characteristics of the Patients

A total of 201 female patients with SLE participated in this study. They were recruited from the Rheumatology, Nephrology, and Dermatology clinics, in 164 (81.59%), 34 (16.91%), and 3 (1.49%) cases, respectively. Demographics and characteristics of the patients studied are shown in Table 1.

Their mean age and median (Q1–Q3) disease duration were 30.75 ± 7.77 years and 9.31 (4.10–14.18) years, respectively. Of the participants, 73.37% of them were single and 26.63% were married or living with their partner. The majority of the patients (85.50%) had a tertiary school level of education. Hypertension and dyslipidemia were common co-morbidities (20.10% in each). None of the patients smoked, and only one currently drank alcohol. During the course of SLE, mucocutaneous, hematological, renal, and musculoskeletal systems were the common organs involved (51.24–83.08%). The patients’ mean number of the 1997 ACR classification criteria and median (Q1–Q3) SDI score was 5.50 ± 1.38 and 0 (0–1), respectively. Their mean cSLEDAI and median (Q1–Q3) cSLEDAI scores were 2.66 ± 4.03 and 0 (0–4), respectively, with 95 patients (47.26%) in DORIS remission. At the time of this survey, renal and mucocutaneous systems were involved in 27.86% and 19.90% of cases, respectively. Prednisolone, hydroxychloroquine, and immunosuppressive drugs were given to 86.06%, 47.00%, and 63.68% of the patients, respectively. A total of 167 patients (83.08%) were hospitalized, 17 (10.08%) were admitted to the intensive care unit, and 117 (70.06%) stayed for more than 5 days.

### 3.2. Patients’ Perceptions of Their Disease Severity

One hundred and sixty-three patients (81.09%) perceived that their SLE disease was severe (Table 2), which was mostly related to renal involvement (55.22%), followed by the musculoskeletal (22.89%), hematologic (21.39%), and nervous systems (12.94%).

### 3.3. Reasons to Avoid Pregnancy

Of the 201 patients, 127 (63.18%) did not desire to become pregnant. Interestingly, 72 (56.69%) made the decision independently. Other main reasons for avoiding pregnancy included fear of worsening SLE flares in 60 patients (47.24%) and bad past experience with the disease also in 60 (47.24%). Thirty-one patients (24.41%) were afraid that the disease and/or medications might affect the fetus, and only 10 (7.87%) were advised by the physician to refrain from pregnancy. A small proportion of the patients (8.66%) had no desire to become pregnant for other reasons such as fear that their baby would develop SLE or an anomaly and not considering pregnancy yet (Table 3).

### 3.4. Factors Associated with the Desire to Avoid Pregnancy

The demographic and clinical characteristics of patients who did and did not want to become pregnant were compared (Supplementary Table S1). Overall, there was no significant difference in demographic data, attendance in subspecialty clinics, educational status, co-morbidities, cumulative clinical manifestations, current organ involvement, treatment received, SLE disease activity, proportion of patients achieving DORIS remission, history of hospitalization, and patients’ perception of severe disease between the 2 groups. However, those avoiding pregnancy were older (31.62 years vs. 29.25 years, *p* = 0.025) and had a lower educational level (primary and secondary school) (19.05% vs. 6.76%, *p* = 0.017). They also had less cumulative mucocutaneous and hematologic involvement (79.53% vs. 89.18%, *p* = 0.078, and 71.65% vs. 83.78%, *p* = 0.052, respectively), but more hospitalization (86.61% vs. 77.03%, *p* = 0.080). In addition, based on the patients’ rating of disease severity, renal involvement was more common in those who did not want to become pregnant than in those who did (59.84% vs. 47.03%, *p* = 0.085).

To identify possible factors associated with the wish to avoid pregnancy, those with a *p*-value of <0.15 in the univariable analysis were included in the multivariable logistic regression analysis. Age was found to be an independent factor associated with the desire to avoid pregnancy, with an adjusted odds ratio and 95% confidence interval (AOR [95% CI]) of 1.07 (1.02–1.11), *p* = 0.005, whereas being married or living with a partner and having a tertiary level of education were independent factors associated with less likelihood of avoiding pregnancy (AOR [95% CI]: 0.39 [0.18–0.77], *p* = 0.008 and 0.29 [0.10–0.81], *p* = 0.018, respectively) (Table 4).

As being single, married, or living with a partner may influence the decision to become pregnant, factors associated with wanting or not wanting to were determined separately in the subgroup of single patients (Supplementary Table S2 and Table 5) and in patients who were married or living with their partner (Supplementary Table S3 and Table 6). There was no significant difference in demographic data, attendance in subspecialty clinics, educational status, co-morbidities, cumulative clinical manifestations, current organ involvement, treatment received, SLE disease activity, proportion of patients achieving DORIS remission, history of hospitalization, and patients’ perception of severe disease between those who did and did not want pregnancy. However, in the regression analysis, longer disease duration among single patients was the only independent factor associated with avoiding pregnancy (AOR [95% CI]: 1.06 [1.00–1.12], *p* = 0.034) (Table 5). Among patients who were married or living with their partner, older age and older age at SLE onset were independent factors associated with not wanting pregnancy (AOR [95% CI]: 1.21 [1.05–1.40], *p* = 0.011, and 1.16 [1.01–1.34], *p* = 0.038, respectively) (Table 6).

## 4. Discussion

This study found that two-thirds of the never-pregnant Thai SLE patients wished to avoid pregnancy. The primary reasons for avoiding it included personal decisions, fear of worsening disease, past negative experiences, and concerns about the effects of the disease or medications on the fetus. Increasing age was an independent factor associated with no desire to become pregnant. In contrast, being married or living with a partner, as well as having a high level of education (tertiary education) were independent factors associated with less likelihood of wanting to avoid pregnancy; in other words, these factors were associated with a greater desire to become pregnant. Longer disease duration among single patients was an independent factor associated with avoiding pregnancy, while among married patients or patients living with a partner, increasing age and higher age at SLE onset were independently associated with no desire to become pregnant. Desire to avoid pregnancy was not associated with SLE disease activity.

Despite studies on pregnancy and SLE being extensively reported, those on the desire to become pregnant in this population are very limited [24,25]. A study in the Netherlands by Blomjous et al. [24] found that among 51 of their 124 childless patients, 31% were directly related to SLE, 21% to personal decision, and 10% to medical advice and medication use. Interestingly, the course of prior pregnancy influenced the decision to refrain from having more children in 20% of the patients. A study in South Korea by Kim et al. [25] found that among 61 of 112 SLE patients who did not want to become pregnant, 31% was for health reasons, 8% for not wanting more children, and 3% because of their economic status. This study found that 63% of the patients did not desire to become pregnant, in which almost 60% was by personal decision, while the remainder was generally related to fear of pregnancy affecting the patients’ health, or medication affecting the fetus. The relatively high prevalence of avoiding pregnancy in this Thai SLE population was not clearly understood when compared to the studies in the Netherlands and South Korea. It might be related to the fact that this study included only never-pregnant SLE patients, as well as differences in ethnicity, culture, and economic status of the study population.

It is interesting that past SLE experience contributed to avoiding pregnancy in almost half of the patients in this study. This number was much higher than that reported from the Netherlands and South Korea [24,25]. Clearly, SLE patients with very active or severe disease are often admitted to hospital in order to control their condition. They often receive treatment with high dose glucocorticosteroids, immunosuppressive drugs, and other medicines, or they may experience complications from treatment. These factors might have discouraged them from becoming pregnant. Unfortunately, to the best of the authors’ knowledge, there has been no study that specifically determines the relationship between hospitalization and desire to become pregnant. This study found that patients avoiding pregnancy were hospitalized more than those who desired to become pregnant, although this did not reach statistical significance.

More than half of the patients in this study did not want to become pregnant due to personal decisions, which is another interesting issue. This prevalence also was higher than that reported from the Netherlands [24]. Unfortunately, this decision was not provided from the study in South Korea [25]. In addition to the health problems mentioned above, the high prevalence of SLE patients avoiding pregnancy might also be related to the worldwide decrease in pregnancy rates [26]. In Thailand, the rate of pregnancy among the general population also has declined over the past decade [27], due to several factors that discourage younger generations from having children, including economic downturns, high parenting costs, debt, and the desire for personal success. These pressures could influence people’s attitudes and perspectives, resulting in a decreased desire to have children [28]. The study in South Korea found that the prevalence of SLE patients desiring to avoid pregnancy was higher than that in the controls [25]. As this study did not have controls, it is unknown whether the prevalence was lower in SLE patients wanting pregnancy than that for the general Thai population.

It was not surprising that being married or living with a male partner was found to be an independent factor associated with a greater desire for pregnancy, as such women are more likely to want children. Also, it is interesting that a high educational level was found to be an independent factor associated with less desire to avoid pregnancy, in other words, a higher educational level was associated with a greater desire to become pregnant, although this could not be confirmed in the subgroup analysis of either those who were single or those who were married or living with their partner. The reason for this finding might be because a higher education enables a higher family income, which makes having children more likely.

As almost three-fourths of the patients in this study were single, they might not currently be planning pregnancy, but they may if they have a partner in the future. Therefore, they should avoid using cyclophosphamide during this period or use it only for a short time if necessary. Reproductive health consultation should be provided [11,12]. Effective contraception methods such as intrauterine devices, vaginal ring, progestin only oral pill or depot-medroxyprogesterone acetate (DMPA) injection are recommended. However, estrogen containing contraceptive pills should be considered only in cases with stable or low disease activity and not positive for antiphospholipid antibodies. In addition, the use of gonadotropin-releasing hormone (GnRH) should be considered for ovarian preservation among menstruating SLE patients who are about to receive alkylating agents [11]. Assisted reproduction techniques could be safe for patients with stable or inactive disease [11].

This study had some limitations. The questionnaire was developed by this study group and, although not formally validated, it was pilot tested with 5 SLE patients and 1 schoolteacher to ensure clarity. It was written in Thai and understandable for the patients in this study. The data on participants’ income were not collected, so the effect of economic status on the desire to avoid pregnancy was not assessable. A study from South Korea found that economic factors clearly influenced the decision to avoid becoming pregnant, which was lower in SLE patients than in the general population [25]. The diagnosis of SLE in this study was based on the 1997 ACR or 2012 SLICC classification criteria. The 2019 ACR/EULAR classification criteria were not used because many patients were diagnosed with SLE before the criteria were introduced [29]. However, the 1997 ACR and the 2012 SLICC classification criteria remain widely accepted for SLE classification. This study included patients taking teratogenic drugs, such as methotrexate, mycophenolate mofetil, and cyclophosphamide, which might have influenced their decision to avoid pregnancy at the time of this survey. Although the proportion of those who avoided pregnancy received more immunosuppressive drugs than those who desired it, this difference did not reach statistical significance. Furthermore, only one-fourth of the patients reported that their decision to avoid pregnancy was due to concerns of SLE medication possibly affecting their baby. This suggests that immunosuppressive drug use was not the primary reason for wanting to avoid pregnancy in this study population. Being a single-center study, its results regarding prevalence, reasons for, and possible factors associated with not wanting pregnancy might not be generalizable, due to differences in ethnicities, cultures, and socioeconomic status. To the best of the authors’ knowledge, there have been no studies in Thailand that determine the reasons for avoiding pregnancy in other rheumatic diseases (such as systemic sclerosis, rheumatoid arthritis, spondyloarthropathies, polymyositis, or vasculitis). Therefore, it is not known whether the prevalence of pregnancy avoidance in these conditions is similar to that observed in SLE.

This study had several strengths. It was conducted among SLE patients who had never been pregnant, thus providing an accurate assessment of the proportion of SLE patients avoiding pregnancy and the reasons for their decision. Understanding why SLE patients of childbearing age do not wish to become pregnant could lead to greater flexibility in managing severe disease, which requires long-term immunosuppressive drugs that are potentially teratogenic (such as methotrexate, mycophenolate mofetil, and cyclophosphamide). This especially applies in economically disadvantaged settings, where newer and potentially safer SLE medications, such as cyclosporine, voclosporin, tacrolimus, belimumab, and anifrolumab, may be unaffordable for patients or unavailable in developing or underdeveloped countries.

## 5. Conclusions

More than half of the never-pregnant Thai SLE patients in this study center desired to avoid pregnancy. Personal decisions and prior experience with their disease or other health issues were the main reasons for this. Increasing age was an independent factor associated with a lack of desire to become pregnant, while being married or living with a partner, as well as having a high level of education (tertiary education), were independent factors associated with less likelihood of avoiding pregnancy. Desire to become pregnant should be discussed with SLE patients during management of the disease. This could lead to greater flexibility in treating active and severe cases, particularly in those who require long-term medications that affect ovarian function.

## Figures and Tables

**Table 1:** Demographic characteristics of the patients studied.

	N = 201
Age (years)	30.75 ± 7.77
Age at SLE onset (years)	20.91 ± 7.00
Disease duration (years)	9.31 (4.10–14.18)
Marital status	
Single	146/199 (73.37)
Living with partner	53/199 (26.63)
Educational status	
Primary/secondary level	29/200 (14.50)
Tertiary level	171/200 (85.50)
Co-morbidities	
Hypertension	41 (20.40)
Diabetes mellitus	2 (1.00)
Dyslipidemia	41 (20.40)
Others *	17 (8.46)
Cumulative manifestation according to 1997 ACR classification criteria	
Mucocutaneous system	167 (83.08)
Musculoskeletal system	103 (51.24)
Cardiopulmonary system	28 (13.93)
Neurological system	26 (12.94)
Hematologic system	153 (76.12)
Renal system	146 (72.64)
Anti-nuclear antibody, n/N (%)	198/199 (99.50)
Immunology, n/N (%)	
Anti-dsDNA antibody, n/N (%)	160/189 (84.66)
Anti-Sm antibody, n/N (%)	9/44 (20.45)
Anti-phospholipid antibodies, ^#^ n/N (%)	11/106 (10.38)
Number of ACR criteria	5.50 ± 1.38
SDI scores	0 (0–1)
Current active organ involvement	
Mucocutaneous system	40 (19.90)
Musculoskeletal system	8 (3.98)
Cardiopulmonary system	1 (0.50)
Neurological system	1 (0.50)
Hematologic system	10 (4.98)
Renal system	56 (27.86)
Current treatment	
Prednisolone	177 (88.06)
Prednisolone dose (mg/day)	5 (5–10)
Hydroxychloroquine	94 (47.00)
Hydroxychloroquine dose (mg/day)	200 (100–200)
Immunosuppressive drugs ^‡^	128 (63.68)
Methotrexate	9 (4.48)
Methotrexate dose (mg/week)	15 (12.50–20.00)
Azathioprine	14 (6.97)
Azathioprine (mg/day)	50 (50–50)
Mycophenolate mofetil	95 (47.26)
Mycophenolate mofetil dose (mg/day)	1000 (500–2000)
Cyclosporine	17 (8.46)
Cyclosporine dose (mg/day)	100 (100–150)
Cyclophosphamide	12 (5.97)
Cyclophosphamide dose (mg/month)	1000 (1000–1500)
SLE disease activity	
cSLEDAI score	2.66 ± 4.03, 0 (0–4)
cSLEDAI = 0	115 (57.21%)
cSLEDAI ≥ 1	86 (42.79%)
DORIS remission	95 (47.26%)
Hospitalization	
Previous Hospitalization	167 (83.08)
Number of hospitalizations	2 (1–5)
Hospitalization > 5 days	117 (70.06)
ICU admission	17 (10.18)
Number of ICU admissions	1 (1–2)

Data are expressed as mean ± SD, median (Q1–Q3) or n (%). n/N = number of positive tests/number of patients tested. * = thalassemia in 6, thyrotoxicosis in 2, renal calculi in 2, myasthenia gravis in 2, ischemic stroke in 2, pulmonary hemosiderosis in 1, history of thyroid carcinoma in 1 and chronic kidney disease in 1. ^#^ = anti-cardiolipin antibody and lupus anti-coagulant. ^‡^ = a patient might receive more than one immunosuppressive drug. ACR = American College of Rheumatology, cSLEDAI = Clinical Systemic Lupus Erythematosus Disease Activity Index, DORIS = Definition of Remission in Systemic Lupus Erythematosus, ICU = intensive care unit, SLE = Systemic lupus erythematosus, SLICC = Systemic Lupus Erythematosus International Collaboration Clinics, SDI = SLICC/ACR Damage Index.

**Table 2:** Patients’ perceptions on severity of their disease.

	N = 201
Patients’ perception of having severe SLE	163 (81.09)
Organ that the patients perceived as severely involved *	
Nervous system	26 (12.94)
Renal system	111 (55.22)
Musculoskeletal system	46 (22.89)
Cardiopulmonary system	19 (9.45)
Hematologic system	43 (21.39)
Mucocutaneous system	8 (3.98)
Gastrointestinal system	1 (0.50)
Number of severe organ involvements	1 (1–2)

Data are expressed as N (%) or median (Q1–Q3). * = a patient might indicate more than one severe organ involvement. SLE = Systemic lupus erythematosus.

**Table 3:** Reasons for not wishing to become pregnant.

	N = 201
Desire to become pregnant	74 (36.82)
Desire to avoid pregnancy	127 (63.18)
Reason for desiring to avoid pregnancy *	N = 127
I really do not want to have a baby	72 (56.69)
I am afraid that pregnancy will cause my disease flares or worsen them	60 (47.24)
I had a bad experience with my disease in the past	60 (47.24)
I am afraid my disease and medication will affect my baby	31 (24.41)
My physician suggests that I should not have a baby	10 (7.87)
Others: (I am afraid that my baby will have SLE, I am sick, I do not think about pregnancy at this moment, I am afraid that my fetus will have anomalies)	11 (8.66)

Data are expressed as N (%). * = more than one response can be applied.

**Table 4:** Logistic regression analysis of the desire to avoid pregnancy (all patients).

	Total Cases	Desire to Avoid Pregnancy, n (%)	Univariate Analysis			Logistic Regression		
			OR	95% CI	*p*-Value	AOR	95% CI	*p*-Value
Age (years)	201	127 (63.18)	1.04	1.00–1.08	0.039	1.07	1.02–1.11	0.005
Disease duration (years)	201	127 (63.18)	1.03	0.99–1.08	0.124			
Marital status								
Single	146	98 (67.12)						
Married or living with partners	53	28 (52.83)	0.55	0.29–1.04	0.066	0.38	0.18–0.77	0.008
Educational status								
Primary/secondary level	29	24 (82.76)						
Tertiary level	171	102 (59.65)	0.31	0.11–0.85	0.022	0.29	0.10–0.81	0.018
Current treatment, immunosuppressive drugs								
No	73	41 (56.16)						
Yes	128	86 (67.19)	1.60	0.89–2.89	0.120			
Previous hospitalization								
No	34	17 (50.00)						
Yes	167	110 (65.87)	1.93	0.92–4.063	0.08			
Organ that the patients perceived as severely involved								
Renal								
No	90	51 (56.67)						
Yes	111	76 (68.47)	1.66	0.93–2.96	0.086			

OR = Odds ratio, AOR = Adjusted odds ratio.

**Table 5:** Logistic regression analysis for the desire to avoid pregnancy (among single patients).

	Total Cases	Desire to Avoid Pregnancy, n (%)	Univariate Analysis			Logistic Regression		
			OR	95% CI	*p*-Value	AOR	95% CI	*p*-Value
Disease duration (years)	146	98 (67.12)	1.06	1.00–1.11	0.049	1.06	1.00–1.12	0.034
Subspecialty clinic								
Rheumatology	119	77 (64.71)						
Non-Rheumatology	27	21 (77.78)	1.91	0.71–5.10	0.197			
Educational status								
Primary/secondary level	22	18 (81.82)						
Tertiary level	124	80 (64.52)	0.40	0.13–1.27	0.121	0.36	0.11–1.14	0.082
Co-morbidities								
Hypertension								
No	116	74 (63.79)						
Yes	30	24 (80.00)	2.27	0.86–6.00	0.098			
Current active organ manifestations								
Renal, n (%)								
No	103	65 (63.11)						
Yes	43	33 (76.74)	1.93	0.86–4.35	0.113			
Current treatment, immunosuppressive drugs								
No	53	31 (58.49)						
Yes	93	67 (72.04)	1.83	0.90–3.72	0.095			
Previous hospitalization								
No	24	13 (54.17)						
Yes	122	85 (69.67)	1.94	0.80–4.74	0.144			
Organ that the patients perceived as severely involved								
Renal								
No	64	39 (60.94)						
Yes	82	59 (71.95)	1.64	0.82–3.30	0.161			

**Table 6:** Logistic regression analysis for the desire to avoid pregnancy (among married patients or patients living with a partner).

	Total Cases	Desire to Avoid Pregnancy, n (%)	Univariate Analysis			Logistic Regression		
			OR	95% CI	*p*-Value	AOR	95% CI	*p*-Value
Age (years)	53	28 (52.83)	1.23	1.09–1.39	0.001	1.21	1.05–1.40	0.011
Age at SLE onset (years)	53	28 (52.83)	1.21	1.07–1.36	0.003	1.16	1.01–1.34	0.038
Subspecialty clinic								
Rheumatology	44	24 (54.55)						
Non-Rheumatology	9	4 (44.44)	0.67	0.16–2.82	0.582			
Educational status								
Primary/secondary level	7	6 (85.71)						
Tertiary level	45	21 (46.67)	0.15	0.02–1.31	0.086	0.62	0.05–7.02	0.696

## Data Availability

The raw data supporting the conclusions of this article will be made available by the authors on request.

## Supplementary Materials

The following supporting information can be downloaded at: https://www.mdpi.com/article/10.3390/jcm14186394/s1, Supplementary Table S1: Comparison between SLE patients who desired to avoid pregnancy and those who desired to become pregnant (all patients); Supplementary Table S2: Comparison between SLE patients who desired to avoid pregnancy and those who desired to become pregnant (single patients); Supplementary Table S3: Comparison between SLE patients who desired to avoid pregnancy and those who desired to become pregnant (married patients or patients living with a partner).

## Abbreviations

The following abbreviations are used in this manuscript:

ACR	American College of Rheumatology
AOR	Adjusted odds ratio
DMPA	Depot medroxyprogesterone acetate
GnRH	Gonadotropin-releasing hormone
SDI	SLICC/ACR Damage Index
SLE	Systemic lupus erythematosus
SLICC	Systemic Lupus Erythematosus International Collaboration Clinics
95%CI	95% confidence interval
