# Supplementary material for: Prevalence and Factors Associated with the Desire to Avoid Pregnancy in Never-Pregnant Patients with Systemic Lupus Erythematosus
Source: J Clin Med. 2025 Sep 10;14(18):6394. doi: 10.3390/jcm14186394 (PMC12470257; doi:10.3390/jcm14186394)
Supplement: Supplementary file 1 [file jcm-14-06394-s001.zip › Supplementary materials (S1)/Supplementary Table S1. Desire vs. avoiding pregnancy (all patients).pdf]

Supplementary Table S1. Comparison between SLE patients who desired to avoid pregnancy and those who desired to become pregnant (all patients)

	All patients (N=201)		
	**Desired to avoid pregnancy (N=127)**	**Desired to become pregnant (N=74)**	**p- value**
Age (years)	31.62±8.40	29.25±6.33	0.025
Age at SLE onset (years)	21.20±7.43	20.41±6.22	0.443
Disease duration (years)	9.38 (4.10-15.01)	7.31 (3.92-12.17)	0.195
Marital status (N=199)			
Single	98/126 (77.78)	48/73 (65.75)	0.064
Living with a partner	28/126 (22.22)	25/73 (34.25)	
Subspecialty clinic			
Rheumatology	101 (79.53)	63 (85.14)	0.322
Non-Rheumatology	26 (20.47)	11 (14.86)	
Educational status			
Primary/secondary level	24 (19.05)	5 (6.76)	0.017
Tertiary level	102 (80.95)	69 (93.24)	
Co-morbidities			
Hypertension	29 (22.83)	12 (16.22)	0.261
Diabetes mellitus	1 (0.79)	1 (1.35)	1.000
Dyslipidemia	26 (20.47)	15 (20.27)	0.973
Others*	14 (11.02)	3 (4.05)	0.087
**Cumulative manifestation according to 1997 ACR classification criteria**			
Mucocutaneous system	101 (79.53)	66 (89.19)	0.078
Musculoskeletal system	62 (48.82)	41 (55.41)	0.368
Cardiopulmonary system	20 (15.75)	8 (10.81)	0.330
Neurological system	15 (11.81)	11 (14.86)	0.534
Hematologic system	91 (71.65)	62 (83.78)	0.052
Renal system	94 (74.02)	52 (70.27)	0.566
Anti-nuclear antibody, n/N (%)	125/125 (100.00)	73 (98.65)	0.372
Immunology, n/N (%)			
Anti-dsDNA antibody, n/N (%)	103/118 (87.29)	57/71 (80.28)	0.196
Anti-Sm antibody, n/N (%)	4/26 (15.38)	5/18 (27.78)	0.316
Anti-phospholipid antibodies,[#] n/N (%)	6/67 (8.96)	5/39 (12.82)	0.529
Number of ACR criteria	5.46±1.42	5.57±1.32)	0.626
SDI scores	0 (0- 1)	0 (0-1)	0.342
**Current active organ manifestations**			
Mucocutaneous system	26 (20.47)	14 (18.92)	0.790
Musculoskeletal system	4 (3.15)	4 (5.41)	0.469
Cardiopulmonary system	1 (0.79)	0 (0.00)	1.000
Neurological system	0 (0.00)	1 (1.35)	0.368
Hematologic system	4 (3.15)	6 (8.11)	0.119
Renal system	39 (30.71)	17 (22.97)	0.238
**Current treatment**			
Prednisolone	112 (88.19)	65 (87.84)	0.941
Hydroxychloroquine	56 (44.09)	38 (51.35)	0.320

Immunosuppressive drugs	86 (67.72)	42 (56.76)	0.119
**Hospitalization**			
Previous hospitalization	110 (86.61)	57 (77.03)	0.080
Number of hospitalizations	2 (1- 6)	2 (1-5)	0.824
Hospitalization >5 days	77/110 (70.00)	40/57 (70.18)	0.981
ICU admission	13/110 (11.82)	4/57 (7.02)	0.331
Number of ICU admission	1 (1-3)	1.5 (1-2)	0.235
**Patients' perception**			
Patients' perception of having severe SLE	107 (84.25)	56 (75.68)	0.134
**Organ that the patients perceived as severely involved**			
Nervous system	17 (13.39)	9 (12.16)	0.803
Renal system	76 (59.84)	35 (47.30)	0.085
Musculoskeletal system	29 (22.83)	17 (22.97)	0.982
Cardiopulmonary system	13 (10.24)	6 (8.11)	0.619
Hematologic system	26 (20.47)	17 (22.97)	0.677
Mucocutaneous system	6 (4.72)	2 (2.70)	0.713
Gastrointestinal system	0	1 (1.35)	0.368
Number of severe organ involvement	1 (1-2)	1 (0-2)	0.293

Data are expressed as mean ± SD, median (p25-p75) or n (%). n/N = number of positive tests or positive responses/number of patients tested or number of respondents. * = thalassemia in 6, thyrotoxicosis in 2, renal calculi in 2, myasthenia gravis in 2, ischemic stroke in 2, pulmonary hemosiderosis in 1, and history of thyroid carcinoma in 1 and chronic kidney disease in 1. # = anti-cardiolipin antibody and lupus anti-coagulant.

ACR = American College of Rheumatology, ICU = intensive care unit, SLE = Systemic lupus erythematosus, SLICC = Systemic Lupus Erythematosus International Collaboration Clinics, SDI = SLICC/ACR Damage Index.
