# Supplementary material for: Prevalence and Factors Associated with the Desire to Avoid Pregnancy in Never-Pregnant Patients with Systemic Lupus Erythematosus
Source: J Clin Med. 2025 Sep 10;14(18):6394. doi: 10.3390/jcm14186394 (PMC12470257; doi:10.3390/jcm14186394)
Supplement: Supplementary file 1 [file jcm-14-06394-s001.zip › Supplementary materials (S1)/Supplementary Table S2. Desire vs. avoiding pregnancy (single patients).pdf]

Supplementary Table S2. Comparison between SLE patients who desired to avoid pregnancy and those who desired to become pregnant (single patients)

	Single patients (N=146)		
	**Desired to avoid pregnancy (N=98)**	**Desired to become pregnant (N=48)**	**p-value**
Age (years)	29.93±8.35	28.33±6.77	0.217
Age at SLE onset (years)	19.24±6.27	20.14±6.84	0.432
Disease duration (years)	9.49 (4.50-15.43)	6.89 (3.66-12.21)	0.069
Subspecialty clinic			
Rheumatology	77 (78.57)	42 (87.50)	0.192
Non-Rheumatology	21 (21.43)	6 (12.50)	
Educational status			
Primary/secondary level	18 (18.37)	4 (8.33)	0.111
Tertiary level	80 (81.63)	44 (91.67)	
Co-morbidities			
Hypertension	24 (24.49)	6 (12.50)	0.092
DM	1 (1.02)	0	1.000
Dyslipidemia	21 (21.43)	10 (20.83)	0.934
Others*	9 (9.18)	2 (4.17)	0.281
**Cumulative manifestation according to 1997 ACR classification criteria**			
Mucocutaneous system	79 (80.61)	44 (91.67)	0.085
Musculoskeletal system	45 (45.92)	28 (58.33)	0.159
Cardiopulmonary system	15 (15.31)	6 (12.50)	0.650
Neurological system	15 (15.31)	8 (16.67)	0.832
Hematologic system	71 (72.45)	41 (85.42)	0.082
Renal system	72 (73.47)	32 (66.67)	0.394
Anti-nuclear antibody, n/N (%)	96/96 (100.00)	47 (97.92)	0.333
Immunology, n/N (%)			
Anti-dsDNA antibody, n/N (%)	80/91 (87.91)	39/48 (81.25)	0.287
Anti-Sm antibody, n/N (%)	3/20 (15.00)	2/11 (18.18)	1.000
Anti-phospholipid antibodies,[#] n/N (%)	6/55 (10.91)	2/23 (8.70)	1.000
Number of ACR criteria	5.48±1.43	5.55±1.38	0.807
SLICC organ damage scores	0 (0-1)	0 (0-1)	0.307
**Current active organ manifestations**			
Mucocutaneous system	20 (20.41)	8 (16.67)	0.590
Musculoskeletal system	3 (3.06)	4 (8.33)	0.218
Cardiopulmonary system	1 (1.02)	0	1.000
Neurological system	0	1 (2.08)	0.329
Hematologic system	3 (3.06)	3 (6.25)	0.395
Renal system	33 (33.67)	10 (20.83)	0.110
**Current treatment**			
Prednisolone	88 (89.80)	43 (89.58)	0.968
Hydroxychloroquine	42 (42.86)	25 (52.08)	0.293
Immunosuppressive drugs	67 (68.37)	26 (54.17)	0.094
**Hospitalization**			
Previous hospitalization	85 (86.73)	37 (77.08)	0.139
Number of hospitalizations	2 (1-6)	2 (1-4)	0.517
Hospitalization >5 days	62/85 (72.94)	25/37 (67.57)	0.546
ICU admission	12/85 (14.12)	3/37 (8.11)	0.550

Number of ICU admission	1 (1-2.5)	2 (1-2)	0.623
**Patients' perception**			
Patients' perception of having severe SLE	84 (85.71)	37 (77.08)	0.193
**Organ that the patients perceived as severely involved**			
Nervous system	16 (16.33)	4 (8.33)	0.187
Renal system	59 (60.20)	23 (47.92)	0.160
Musculoskeletal system	24 (24.49)	10 (20.83)	0.623
Cardiopulmonary system	9 (9.18)	4 (8.33)	0.865
Hematologic system	21 (21.43)	11 (22.92)	0.838
Mucocutaneous system	4 (4.08)	0	0.303
Gastrointestinal system	0	1 (2.08)	0.329
Number of severe organ involvement	1 (1-2)	1 (0.5-2)	0.186

Data are expressed as mean ± SD, median (p25-p75) or n (%). n/N = number of positive tests or positive responses/number of patients tested or number of respondents. * = thalassemia in 5, thyrotoxicosis in 1, panniculitis in 1, myasthenia gravis in 1, idiopathic pulmonary hemosiderosis in 1, and history of thyroid carcinoma in 1 and chronic kidney disease in 1. # = anti-cardiolipin antibody and lupus anti-coagulant.

ACR = American College of Rheumatology, ANA = anti-nuclear antibody, Anti-dsDNA = anti-double stranded DNA antibody, anti-Sm = anti-Smith antibody, ICU = intensive care unit, SLE = Systemic lupus erythematosus, SLICC = Systemic Lupus Erythematosus International Collaboration Clinics, SDI = SLICC/ACR Damage Index.
