# Supplementary material for: Prevalence and Factors Associated with the Desire to Avoid Pregnancy in Never-Pregnant Patients with Systemic Lupus Erythematosus
Source: J Clin Med. 2025 Sep 10;14(18):6394. doi: 10.3390/jcm14186394 (PMC12470257; doi:10.3390/jcm14186394)
Supplement: Supplementary file 1 [file jcm-14-06394-s001.zip › Supplementary materials (S1)/Supplementary Table S3. Desire vs. avoiding pregnancy (married patients).pdf]

**Supplementary Table S3.** Comparison between SLE patients who desired to avoid pregnancy and those who desired to become pregnant (married patients or patients living with a partner)

| | **Married or living with a partner (N=53)** | | |
|---|---|---|---|
| | **Desired to avoid pregnancy (N= 28)** | **Desired to become pregnant (N=25)** | **p-value** |
| Age (years) | 37.34±5.82 | 31.00±5.22 | <0.001 |
| Age at SLE onset (years) | 27.76±7.43 | 21.00±5.05 | <0.001 |
| Disease duration (years) | 8.17 (2.91-14.64) | 10.57 (5.93-12.17) | 0.708 |
| Subspecialty clinic | | | |
| Rheumatology | 24 (85.71) | 20 (80.00) | 0.580 |
| Non-Rheumatology | 4 (14.29) | 5 (20.00) | |
| Educational status | | | |
| Primary/secondary level | 6 (22.22) | 1 (4.00) | 0.054 |
| Tertiary level | 21 (77.78) | 24 (96.00) | |
| Co-morbidities | | | |
| Hypertension | 5 (17.86) | 6 (24.00) | 0.582 |
| DM | 0 | 1 (4.00) | 0.472 |
| Dyslipidemia | 5 (17.86) | 5 (20.00) | 0.842 |
| Others* | 5 (17.85) | 1 (4.00) | 0.112 |
| **Cumulative manifestation according to 1997 ACR classification criteria** | | | |
| Mucocutaneous system | 21 (75.00) | 21 (84.00) | 0.420 |
| Musculoskeletal system | 17 (60.71) | 12 (48.00) | 0.353 |
| Cardiopulmonary system | 5 (17.86) | 2 (8.00) | 0.290 |
| Neurological system | 0 | 3 (12.00) | 0.098 |
| Hematologic system | 19 (67.86) | 20 (80.00) | 0.317 |
| Renal system | 21 (75.00) | 19 (76.00) | 0.933 |
| Anti-nuclear antibody, n/N (%) | 28 (100.00) | 25 (100.00) | |
| Immunology, n/N (%) | | | |
| Anti-dsDNA antibody, n/N (%) | 22/26 (84.62) | 18/22 (81.82) | 1.000 |
| Anti-Sm antibody, n/N (%) | 1/6 (16.67) | 3/7 (42.86) | 0.559 |
| Anti-phospholipid antibodies,[#] n/N (%) | 0/12 (0.00) | 3/15 (20.00) | 0.231 |
| Mean ACR criteria | 5.39±1.41 | 5.61±1.20 | 0.599 |
| SDI scores | 0 (0-1) | 0 (0-1) | 0.923 |
| **Current active organ manifestations** | | | |
| Mucocutaneous system | 6 (21.43) | 6 (24.00) | 0.823 |
| Musculoskeletal system | 1 (3.57) | 0 (0.00) | 1.000 |
| Cardiopulmonary system | 0 | 0 | |
| Neurological system | 0 | 0 | |
| Hematologic system | 1 (3.57) | 3 (12.00) | 0.333 |
| Renal system | 6 (21.43) | 6 (24.00) | 0.823 |
| **Current treatment** | | | |
| Prednisolone | 24 (85.71) | 21 (84.00) | 1.000 |
| Hydroxychloroquine | 14 (50.00) | 13 (54.17) | 0.764 |
| Immunosuppressive drugs | 19 (67.86) | 15 (60.00) | 0.552 |
| **Hospitalization** | | | |
| Previous hospitalization | 24 (85.71) | 19 (76.00) | 0.367 |
| Number of hospitalizations | 2 (1-4) | 3 (2-7) | 0.140 |
| Hospitalization >5 days | 15/24 (62.50) | 14/19 (73.68) | 0.437 |
| ICU admission | 1/24 (4.17) | 1/19 (5.26) | 1.000 |
| Number of ICU admission | 3 (3-3) | 1 (1-1) | 0.317 |

| | | | |
|---|---|---|---|
| **Patients' perception** | | | |
| Patients' perception of having severe SLE | 22 (78.57) | 19 (76.00) | 0.823 |
| **Organ that the patients perceived as severely involved** | | | |
| Nervous system | 1 (3.57) | 5 (20.00) | 0.060 |
| Renal system | 16 (57.14) | 12 (48.00) | 0.506 |
| Musculoskeletal system | 5 (17.86) | 7 (28.00) | 0.378 |
| Cardiopulmonary system | 4 (14.29) | 2 (8.00) | 0.672 |
| Hematological system | 5 (17.86) | 6 (24.00) | 0.582 |
| Mucocutaneous system | 2 (7.14) | 2 (8.00) | 1.000 |
| Gastrointestinal system | 0 | 0 | |
| Mean number of severe organ involvement | 1 (0.50-1.50) | 1 (1-2) | 0.647 |

Data are expressed as mean ± SD, median (p25-p75) or n (%). n/N = number of positive tests/number of patients tested. * = renal calculi in 2, thalassemia in 1, thyrotoxicosis in 1, myasthenia gravis in 1, and ischemic stroke in 1. # = anti-cardiolipin antibody and lupus anti-coagulant.

ACR = American College of Rheumatology, ICU = intensive care unit, SLE = Systemic lupus erythematosus, SLICC = Systemic Lupus Erythematosus International Collaboration Clinics, SDI = SLICC/ACR Damage Index.
